# Combined Epidemiologic and Entomologic Survey to Detect Urban Malaria Transmission, Guinea, 2018

**DOI:** 10.3201/eid2702.191701

**Published:** 2021-02

**Authors:** Dean Sayre, Alioune Camara, Yaya Barry, Touré Babacar Deen, Denka Camara, Mohamed Dioubaté, Ibrahima Camara, Kalil Keita, Nouman Diakité, Youssoufa Lo, Ibrahima Bah, Hadja Fanta Camara, Mohamed Saran Condé, Aissata Fofana, Abdoulaye Sarr, Eugène Lama, Seth Irish, Mateusz Plucinski

**Affiliations:** Centers for Disease Control and Prevention, Atlanta, Georgia, USA (D. Sayre, A. Sarr, S. Irish, M. Plucinski);; National Malaria Control Program, Conakry, Guinea (A. Camara, Y. Barry, T.B. Deen, D. Camara, M. Dioubaté, I. Camara, K. Keita, N. Diakité, Y. Lo, E. Lama);; Catholic Relief Services, Conakry (I. Bah);; Stop Palu+, Conakry (H.F. Camara, M.S. Condé, A. Fofana);; Centers for Disease Control and Prevention, Conakry (A.Sarr)

**Keywords:** malaria, Anopheles gambiae, mosquito, Plasmodium falciparum, parasites, Guinea, sub-Saharan Africa, vector-borne infections, transmission, urban, epidemiology, entomology, Conakry

## Abstract

Malaria incidence is generally lower in cities than rural areas. However, reported urban malaria incidence may not accurately reflect the level of ongoing transmission, which has potentially large implications for prevention efforts. To guide mosquito net distribution, we assessed the extent of malaria transmission in Conakry, Guinea, in 2018. We found evidence of active malaria transmission.

Vector control strategies are an important tool for the reduction of malaria burden worldwide. However, these strategies, such as the distribution of mosquito nets (long-lasting insecticidal nets [LLINs]), are effective only in settings of ongoing malaria transmission. Malaria transmission is generally lower in urban areas compared with rural ones (*1*,*2*). Moreover, due to population mobility and increased urban access to medical services, malaria cases reported from cities may capture at least some infections acquired in the outlying rural areas, complicating use of incidence data to determine the need for LLINs in urban areas. To guide a recent LLIN distribution campaign, we rapidly assessed malaria transmission in Conakry, Guinea, using a combined epidemiologic and entomologic approach. 

## The Study

During November 19–December 24, 2018, we conducted community and health facility cross-sectional surveys describing key malaria epidemiologic and entomologic indicators in 10 nonadjacent sites in Conakry, Guinea, by using the methods described by Camara et al. (*3*). In addition, a sample of outpatients seeking medical attention were tested for malaria at 25 healthcare facilities across Conakry and asked about recent travel outside of the city (Appendix).

**Figure 1:**
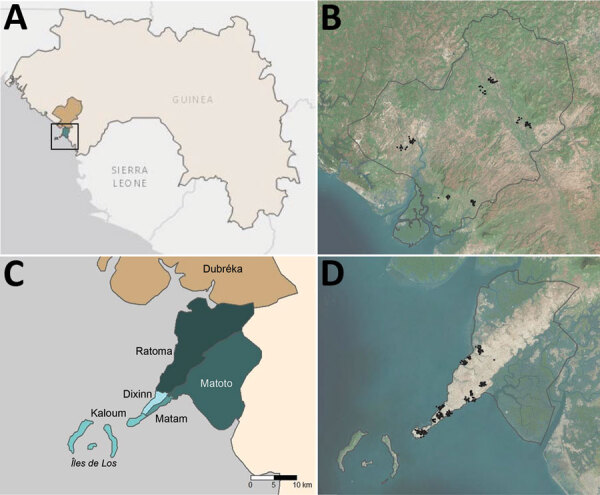
Administrative boundaries and location of communities visited as part of an epidemiologic–entomologic survey in Conakry and Dubréka, 2018. A) The positions of study sites are shown within the context of West Africa specifically Guinea. Turquoise area denotes Conakry and khaki Dubréka. Black square represents boundaries of the area depicted in panel B. B) Satellite imagery of Dubréka (outlined in black) and surrounding areas, with participating households shown as black points. C) Locations of 5 administrative communes within Conakry are shown in shades of turquoise; Dubréka is colored khaki. D) Satellite imagery of Conakry (outlined in black) and surrounding areas, with households participating in the survey shown as black points. Global positioning system coordinates of households were jittered for confidentiality before mapping in both (B) and (D).

We conducted a community survey in 300 households throughout Conakry, yielding person-specific data from 2,164 persons and mosquito net access and use data for 1,016 unique sleeping spaces (Figure 1; Appendix Table 1). We performed rapid diagnostic tests (RDTs) to detect *Plasmodium falciparum*−specific antigens for 1,102 (50.9%) of these persons. Surveys conducted in 120 households in 4 villages within the neighboring rural district of Dubréka provided person-specific data for 919 participants and mosquito net access and use data for 486 unique sleeping spaces to serve as a control. We tested 451 (49.1%) control participants for malaria by RDT.

In Conakry, 43.3% of households surveyed claimed to own >1 mosquito net, compared with 89.2% (p<0.001) of households in Dubréka. Survey participants reported 18.8% (191/1,016) of documented sleeping spaces in Conakry had a mosquito net available at the time of the survey, compared with 63.8% (310/486, p<0.001) in Dubréka. Nets were hanging over 16.7% (170/1,016) of sleeping spaces in Conakry and 59.9% (291/486, p<0.001) of those in Dubréka. However, participant use of nets was similar; 89.0% (170/191) of the available nets in Conakry were in use at the time of the survey, compared with 93.8% (291/310, p = 0.062) in Dubréka (Table 1).

**Table 1:** Coverage of malaria prevention interventions in and near Conakry, Guinea, 2018*

Characteristic	Conakry, no./total no. (%)							Dubréka	p value‡
	Kaloum	Dixinn	Matam	Matoto	Ratoma	Total	p value†		
LLIN ownership									
Households receiving LLIN in last campaign	50/60 (83.3)	48/60 (80.0)	54/60 (90.0)	52/60 (86.7)	52/60 (86.7)	256/300 (85.3)	0.61	102/120 (85.0)	1
Households with >1 LLIN at time of study	14/60 (23.3)	22/60 (36.7)	38/60 (63.3)	24/60 (40.0)	32/60 (53.3)	130/300 (43.3)	<0.001	107/120 (89.2)	<0.001
LLIN access									
Sleeping spaces with LLIN available	20/173 (11.6)	28/169 (16.6)	54/248 (21.8)	26/206 (12.6)	63/220 (28.6)	191/1,016 (18.8)	<0.001	310/486 (63.8)	<0.001
Population sleeping in space with LLIN available	42/366 (11.5)	66/383 (17.2)	108/485 (22.3)	63/445 (14.2)	158/511 (30.9)	437/2,190 (20.0)	<0.001	647/966 (67.0)	<0.001
LLIN use									
Sleeping spaces with LLIN hanging	13/173 (7.5)	26/169 (15.4)	45/248 (18.1)	26/206 (12.6)	60/220 (27.2)	170/1,016 (16.7)	<0.001	291/486 (59.9)	<0.001
Population sleeping in spaces with LLIN hanging	27/366 (7.3)	62/383 (16.2)	89/485 (18.4)	63/445 (14.2)	146/511 (28.6)	387/2,190 (17.3)	<0.001	617/966 (63.9)	<0.001
Spaces with LLIN hanging among those where available	13/20 (65.0)	26/28 (92.9)	45/54 (83.3)	26/26 (100.0)	60/63 (95.2)	170/191 (89.0)	<0.001	291/310 (93.9)	0.062
Proportion sleeping under LLIN in population with access	27/42 (64.3)	62/66 (93.9)	89/108 (82.4)	63/63 (100.0)	146/158 (92.4)	387/437 (88.6)	<0.001	617/647 (95.4)	<0.001
Used LLIN in previous night, <5 y	5/52 (19.6)	19/78 (24.4)	21/99 (21.2)	9/77 (11.7)	24/86 (27.9)	78/392 (19.9)	0.021	137/204 (67.2)	<0.001
Used LLIN in previous night, >5 y	14/314 (4.5)	44/298 (14.8)	99/381 (26.0)	40/371 (10.8)	95/408 (23.3)	292/1,772 (16.5)	<0.001	442/716 (61.7)	<0.001
Indoor residual spraying	4/60 (6.7)	1/60 (1.7)	0/60 (0.0)	1/60 (1.7)	0/60 (0.0)	6/300 (2.0)	0.11	1/120 (0.8)	0.68
Any insecticide use	6/60 (10.0)	9/60 (15.0)	10/60 (16.7)	16/60 (26.7)	20/60 (33.3)	61/300 (20.3)	0.012	54/120 (45.0)	<0.001

*Populations determined by summing responses to number of persons sleeping in each space; total denominators do not necessarily match total number of persons reported as living in surveyed households. LLIN, long-lasting insecticidal nets. †Comparison of results for communes within Conakry. ‡Comparison of results for Conakry and Dubréka.

Mosquito net access and rates of use were found to be heterogeneous across Conakry. Availability of dedicated mosquito nets ranged from 11.6% (20/173) to 28.6% (63/220) of sleeping spaces when households were grouped by administrative sections (communes). Net use when available ranged from 65.0% (13/20) to 100% (26/26) by commune across Conakry (Table 1).

**Figure 2:**
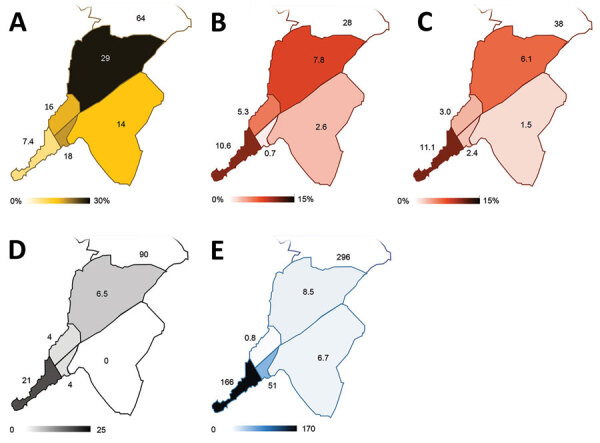
Spatial distribution of key malaria indices across Conakry, Guinea, as assessed during the rapid epidemiologic–entomologic investigation, November–December 2018. Indices are shown grouped by commune within Conakry. A) Proportion of the population reporting sleeping in a space with a mosquito net hanging. B) Malaria prevalence (%) in the sample population >5 years of age, as determined by RDT during community survey. C) Malaria prevalence (%) in the sample population <5 years of age, as determined by RDT during community survey. D) Number of adult female *Anopheles* mosquitoes collected per night, averaged over 2 nights of collection. E) Annualized malaria incidence, reported as cases/1,000 population, diagnosed in local healthcare facilities. RDT, rapid diagnostic test.

Malaria prevalence by RDT in both children <5 years and participants >5 years was lower in Conakry than in Dubréka (Appendix Table 2). RDT positivity among children <5 years was 4.3% (14/329) in Conakry and 38.0% (60/158) in Dubréka (p<0.001); in older participants positivity was 5.6% (43/773) in Conakry and 28.0% (82/293) in Dubréka (p<0.001). Within Conakry, the greatest malaria prevalence in both age groups collocated with the lowest rates of mosquito net use and access, although the differences observed between communes in the younger age group failed to reach statistical significance (p = 0.125 for age <5 years, p<0.001 for those >5 years) (Figure 2). Most participants tested in Conakry (717/1,102, 65.1%) denied having left the city within the last year. Considering only those reporting not having left the city in the past year, we found that 4.0% (29/717) were positive for *P. falciparum* antigen.

Of the 57 participants of all ages who were positive for malaria within Conakry, 75.4% (43/57) reported not leaving the city within the last 4 weeks (Table 2). Thirty-four of these participants (34/57, 59.6%) reported not having left Conakry within the 6 months before interview, and 50.9% (29/57) did not leave the city within the year before interview. Nearly one fifth of Conakry residents were positive for malaria reported never having left the city (17.5%, 10/57).

**Table 2:** Characteristics of persons testing positive for malaria infection, Guinea, 2018*

Characteristic, n/N (%)	Conakry						p value†	Dubréka	p value‡
	Kaloum	Dixinn	Matam	Matoto	Ratoma	Total			
LLIN use previous night, <5 y	0/5 (0.0)	0/2 (0.0)	0/2 (0.0)	1/1 (100)	0/4 (0.0)	1/14 (7.1)	0.07	41/60 (68.3)	<0.001
LLIN use previous night, >5 y	0/17 (0.0)	0/8 (0.0)	0/1 (0.0)	1/4 (25.0)	4/13 (30.8)	5/43 (11.6)	0.042	46/82 (56.1)	<0.001
Fever <2 wk of RDT	15/22 (68.2)	3/10 (30.0)	2/3 (66.7)	5/5 (100)	9/17 (52.9)	34/57 (59.6)	0.092	75/135 (55.6)	0.64
History of travel outside Conakry, <4 wks	4/22 (18.2)	5/10 (50.0)	0/3 (0.0)	1/5 (20.0)	4/17 (23.5)	14/57 (24.6)	0.37	NA	
History of travel outside Conakry, <6 mo	5/22 (22.7)	7/10 (70.0)	0/3 (0.0)	2/5 (40.0)	9/17 (52.9)	23/57 (40.4)	0.04	NA	
No travel outside Conakry within last year	17/22 (77.3)	2/10 (20.0)	1/3 (33.3)	2/5 (40.0)	7/17 (41.2)	29/57 (50.9)	0.016	NA	

*In a joint epidemiologic–entomologic investigation of urban malaria transmission in Guinea, participants were tested by using rapid diagnostic test during community screening. LLIN, long-lasting insecticidal net; NA, not applicable; RDT, rapid diagnostic test. †Comparison of results for communes within Conakry. ‡Comparison of results for Conakry and Dubréka.

A random intercept, mixed effects regression model to identify risk factors for *P. falciparum* antigenemia demonstrated statistically significant associations with self-reported travel outside the city (p<0.23). Odds ratios were 2.2−7.3 and were higher for more recent travel (Appendix Table 3).

We collected recent travel history data from 4,678 persons seeking medical attention whose diagnostic workup included malaria testing by microscopy or RDT. Of these persons, 8.0% (376/4,678) reported travel outside Conakry within the 4 weeks before being tested. Malaria antigen was detected in 57.7% (217/376) of those reporting having left the city in the last 4 weeks, compared with 26.5% (1,139/4,302) of those who remained in Conakry in the same period (Appendix Table 4). The overall relative risk for malaria positivity associated with travel outside of Conakry within the last 4 weeks was 2.2 (95% CI 2.0–2.4). The corresponding population-attributable risk of travel outside the city was calculated as 8.7%. Although rates of malaria positivity and recent travel history both showed large variation, associated relative risks for individual communes were 1.56–3.57 and population-attributable fractions of risk were 4.6%–17.0% across different communes in Conakry.

Collection of adult mosquitoes as part of the study demonstrated the presence of female *Anopheles gambiae* sensu lato mosquitoes in 4/5 communes in Conakry. We captured an average of 21 adult female *A. gambiae* sl. mosquitoes nightly at the urban site yielding the greatest number of *Anopheles* mosquitoes in Conakry (Figure 2; Appendix Figure 1). In contrast, adult mosquito collection from 2 rural sites in Dubréka yielded an average of 90 female *Anopheles gambiae* sensu lato mosquitoes captured per night. However, the nightly yield was highly heterogeneous by site, with 1 of the 2 sites accounting for 99.4% (358/360) of the female *Anopheles* mosquitoes captured (Appendix Figure 2).

## Conclusions

We found multiple corroborating lines of evidence that strongly indicate malaria is actively transmitted in Conakry. The presence of *Anopheles* vectors, current or recent malaria infections in the absence of any plausible travel-related exposures, and the spatial distribution of infection mirroring that of risk factors for local acquisition of disease all suggest ongoing malaria transmission. Rural control sites had greater observed densities of competent vectors and higher prevalence of malaria. In addition, travel outside of the city was found to be a risk for malaria infection for persons living in Conakry. However, we found that the risk associated with travel was a minor contributor to the overall malaria burden in Conakry, indicating that residents appear to be at risk, albeit a decreased one, of acquiring malaria within the confines of the city.

Given the likely ongoing malaria transmission, coupled with the high rate of net use when available, LLIN distribution is a suitable malaria control strategy in Conakry. The observed heterogeneity of malaria transmission across the city raises the potential for more targeted distribution of prevention commodities. Additional studies are needed to confirm and further refine this finding. 

Appendix Additional information about epidemiologic–entomologic survey to detect urban malaria transmission in Guinea, 2018.

## References

[1] De Silva PM, Marshall JM. Factors contributing to urban malaria transmission in sub-saharan Africa: a systematic review. J Trop Med. 2012;2012:819563. 10.1155/2012/819563 23125863 PMC3483782

[2] Pond BS. Malaria indicator surveys demonstrate a markedly lower prevalence of malaria in large cities of sub-Saharan Africa. Malar J. 2013;12:313. 10.1186/1475-2875-12-313 24021162 PMC3848558

[3] Camara A, Guilavogui T, Keita K, Dioubaté M, Barry Y, Camara D, et al. Rapid epidemiological and entomological survey for validation of reported indicators and characterization of local malaria transmission in Guinea, 2017. Am J Trop Med Hyg. 2018;99:1134–44. 10.4269/ajtmh.18-0479 30141394 PMC6221235

