# Supplementary material for: Combined Epidemiologic and Entomologic Survey to Detect Urban Malaria Transmission, Guinea, 2018
Source: Emerg Infect Dis. 2021 Feb;27(2):599–602. doi: 10.3201/eid2702.191701 (PMC7853535; doi:10.3201/eid2702.191701)
Supplement: Appendix — Additional information about epidemiologic–entomologic survey to detect urban malaria transmission in Guinea, 2018. [file 19-1701-Techapp-s1.pdf]

Article DOI: https://doi.org/10.3201/eid2702.191701

Combined Epidemiologic and Entomologic Survey to Detect Urban Malaria Transmission, Guinea, 2018

Appendix

Additional Contextual Information

Conakry is Guinea's largest city with an estimated population of 1.8 million in 2016 (*1*). The city has 5 administrative communes (Figure 1), which vary in size and population (ranging from 70,000–700,000) (*1*).

Although national household surveys demonstrated the overall prevalence of malaria among children <5 years of age to be 44% in 2012 and 30% in 2016, prevalence in Conakry was 3% in 2012 and 2% in 2016 (*2*,*3*). Surveillance of incident malaria cases in Conakry reveals a similar overall pattern; estimated annual incidence for all ages was 18 confirmed cases/1,000 inhabitants. In contrast, the more rural area directly neighboring the city had a recently documented prevalence of 10% (*3*) and incidence of 169 cases/1,000 residents (*4*). All reported cases of malaria from Guinea to the World Health Organization have been due to *Plasmodium falciparum* infection for several years (*5*).

The National Malaria Control Program (NMCP) in Guinea distributes LLINs nationally on a triennial schedule. Previous distribution campaigns were held in in 2013 and 2016. Prior to the LLIN distribution campaign in 2019, we sought to rapidly assess malaria transmission in Conakry to ensure nets would be effective. Definitive proof of malaria transmission often relies on demonstration of *Plasmodium*-infected mosquitoes (*6*). However, definitively ascertaining transmission using purely entomological means requires large samples of *Anopheles* mosquitoes captured in the city and often over long periods (*7–12*). Similarly, most previously published epidemiological methods to gauge urban malaria transmission were also done over longer periods (*12–20*). A series of rapid investigations of urban malaria epidemiology in a select number of cities have been published previously, but these do not address the potential discrepancy between prevalence and active transmission (*21–27*). Using a combined approached, we sought to provide actional information on urban malaria transmission in a timely fashion to inform a rapidly approach LLIN distribution campaign.

Methods

Study Site, Population, and Sampling Approach

All 5 communes of Conakry were entirely included in the consideration for study site selection with the exception of 3 islands off the coast, which are technically part of the city but rural in nature. Routine malaria surveillance data from July–September 2018 were reviewed to identify the areas within each commune reporting the highest malaria incidences. Using these data, 2 geographically separate facility catchment areas were chosen within each of the city's 5 communes (10 urban areas overall) on the basis of elevated malaria incidence as well as input from local partners regarding feasibility and logistic concerns. Each catchment area selected served to provide the sampling units for all phases of the study involving community sampling (households, health facilities, and environmental evaluation for vectors). A similar procedure was conducted for the selection of 4 rural control sites within the rural prefecture of Dubréka just outside of Conakry.

At all chosen healthcare facilities, potential neighborhoods for household surveys were identified by soliciting input from healthcare workers and clinic officials to determine the specific locations contributing the most cases of malaria within the catchment area. The choice of final locations for study were the results of open discussions held between investigators and clinical staff, taking into account malarial disease burden, individual neighborhood accessibility, and overall feasibility.

Household Surveys

Within each study site, investigators randomly selected 30 households using a modified sampling method based on the WHO expanded program on immunization methodology (*28*). Surveys were administered at every third household. At each household, investigators used standardized questionnaires to collect information from the self-identifying head of household. Another adult member of the household was interviewed in lieu of the head of household if needed. All data, including number of household mosquito nets, number of nets hanging, and number of nets in good repair, were collected by participant response rather than surveyor observation. Surveys were designed to assess risk factors for local acquisition of disease as well as travel patterns of Conakry residents. Surveys were conducted and data were collected using electronic tablets loaded with SurveyCTO software version 2.50 (Dobility, https://www.dobility.com).

A maximum of 4 members per household were tested for the presence of *Plasmodium falciparum*–specific histidine-rich protein 2 (HRP2) antigen in the blood using rapid diagnostic tests (RDTs) (SD Bioline Malaria Ag P.f,; Abbott Laboratories, https://www.abbott.com). Children <5 years

were prioritized for testing in order to participants with a limited travel history outside the city. If fewer than 4 children < 5 years were present in the household, testing was offered to other household members, first by offering testing to pregnant women, followed by randomization of older children and non-pregnant adults. If more than 4 children <5 years of age were present, 4 of them were chosen at random for screening. All individuals found to be positive for *P. falciparum* by RDT were given artemisinin-based combination therapy and instructions for use, in accordance with national guidelines.

Healthcare Facility Visits

Registers and routine surveillance reporting forms from each selected healthcare facility were reviewed. Investigators reviewed clinical registers from July–September 2018 to enumerate all-cause consultations, cases of febrile illness (defined as documented subjective fever and or temperature $\geq$38.5° C), cases of confirmed malaria, cases of malaria meeting WHO criteria of severity (Appendix Table 5) (*29*), and malaria-attributable deaths (death in the setting of documented severe malaria in the absence of alternative identified cause). These figures were compared to those reported to the NMCP as part of the monthly national malaria surveillance program.

Travel-Related Risk in Outpatients

Data regarding potential travel-related exposures were collected from persons seeking healthcare throughout Conakry from December 8–27, 2018. All patients visiting participating outpatient facilities for medical attention whose clinical workup included malaria testing were asked to disclose any travel outside the city within the 4 weeks before illness onset. Each facility collected data for 2 weeks using standardized paper forms. Clinical testing was performed in each facility according to its own diagnostic protocols and included a mix of microscopy and RDT. Data were subsequently transcribed to Microsoft Excel 2016 (Microsoft Corporation, https://www.microsoft.com) data files for analysis.

Potential Larval Habitat Enumeration

Mosquito larval surveys were conducted in Conakry by performing exhaustive searches for all accessible pools of standing water within delineated areas of roughly 50,000–90,000 m^2. Search areas were chosen by soliciting healthcare worker and resident opinions on the area within each targeted neighborhood most heavily infested with mosquitoes, a method previously documented to be effective by Mwangungulu et al. (*30*) One survey field was defined for each commune of the city, resulting in a total of 5 urban sites for larval characterization (Appendix Figure 3).

Larval habitat surveys in Dubréka were performed using defined straight line transects to visualize all sources of standing water along the long axis of selected villages rather than within predefined areas. Subsequent, shorter transects were performed in each village at right angles to the

initial path. Two of the 4 villages from Dubréka included in the overall study were chosen for potential larval habitat survey and characterization (Appendix Figure 4).

In all study locations, all collections of standing water were examined for the presence of mosquito larvae. Any mosquito larvae found were classified to the genus level by entomologists. Larvae were not quantified in either relative or absolute terms. Data were collected and stored on electronic tablets loaded with SurveyCTO software.

Adult Mosquito Collection

Adult mosquitoes were captured by human landing catches within each search area selected for larval habitat characterization. Mosquito collections took place continuously from 6:00 PM–7 AM on 2 consecutive nights at each study location. Collections were performed by 2 volunteers working simultaneously, 1 collecting mosquitoes outdoors and the other collecting mosquitoes inside the household of a neighborhood/village resident. Collectors were provided antimalarial prophylaxis free of charge. Adult mosquito collection sites were selected by entomologists to maximize potential nightly yield.

Adult mosquitoes were segregated by location of collection (indoors vs. outdoors) and hour of collection. All mosquitoes were subsequently sexed and morphologically identified to genus. *Anopheles* mosquitoes were morphologically identified to the species/group level.

Data Analysis

Data collected from the household surveys, healthcare facility visits, and entomological studies were subsequently analyzed using R version 3.5.0 (R Foundation for Statistical Computing, www.r-project.org). Data from each commune of Conakry were both statistically compared to one another and aggregated to compare Conakry as a whole against the rural control sites. Fischer's exact test was used for comparison of categorical variables, using an α of 0.05 modified using Bonferroni's correction for multiple comparisons when needed. Factors found to be statistically significant under the assumption of independence (i.e., no clustering) were used to build single variable mixed effects, random intercept models to assess the vigor of statistical significance while accounting for the spatial clustering of data. Household survey data were additionally used to model *P. falciparum* antigenemia using generalized linear mixed effect models using the lme4 package in R (https://github.com/lme4/lme4). Predictors included in the multivariable, random intercept model were respondent age, city commune of residence, travel history, pregnancy status, and self-reported insecticide-treated net use.

Data collected from outpatient visits were used to calculate relative risks, 95% CI, and population-attributable fractions for potential travel-related exposures using Excel. Reported population attributable fractions were calculated using the formula $(I_P - I_u)/I_P$, where I_P = total incidence of malaria at each health facility and I_U = incidence of malaria among non-travelers at each health facility.

Maps for visualization of epidemiological data were generated using tmap package in R (https://github.com/mtennekes/tmap) with shapefiles acquired from GADM (https://gadm.org/download_country_v3.html) and the Humanitarian Data Exchange (https://data.humdata.org/dataset/guinea-geodatabase).

Ethical Considerations

Verbal informed consent was obtained from all participants of this study. Written informed consent form was obtained for each of those participating in the household survey and malaria prevalence screen by RDT. The signature of a parent or guardian was obtained for all children <18 years of age tested by RDT. The protocol for this study was reviewed and approved as a non-research activity by the Center for Global Health’s Office of the Director at the Centers for Disease Control and Prevention (#2017-347a), as well as the Guinea Ministry of Health.

Additional Results

Febrile Illness and Health-Care Seeking Behavior

Rates of subjective fever during the 2 weeks prior to interview were slightly lower in Conakry (18.6%, 398/2,138) than in Dubréka (24.4%, 109/813, p<0.001) (Appendix Figure 5). Reported fever within Conakry also varied by commune, from 15.2% (74/486) to 24.6% (89/362), although these differences did not attain statistical significance when accounting for multiple comparisons using Bonferroni corrections (p = 0.0185).

Healthcare-seeking behavior for febrile illnesses was largely similar across all study sites (Appendix Figure 6). Use of private medical facilities was more common in Conakry (16.7%, 67/402) than in Dubréka (3.0%, 6/198; p<0.001). However, there were no other significant differences in utilization of healthcare facilities by type, including public facilities. Of those experiencing febrile illness, 29.4% (118/402) sought care at a public health facility in Conakry compared to 32.3% (64/198) in Dubréka (p = 0.51). The variations in healthcare-seeking behavior observed across communes of Conakry did not attain statistical significance after accounting for multiple comparisons.

Health Facility Record Review

The facilities in Conakry exhibited variable utilization rates during this time, as evidenced by annualized all-cause incidences ranging from 13–445 consultations/1,000 individuals (Figure 2; Appendix Table 6). One facility visited in the commune of Dixinn serves as a national referral center with a reported catchment population of 12 million people. Although available to the entire population under extraordinary medical circumstances, this reported catchment artificially drives down reported incidences, and as such, the results from this institution are not included in this discussion. Malaria accounted for 15%–71% of all medical consultations among the facilities visited in Conakry.

Compared with the results seen in Conakry, the reported incidences of malaria, all-cause consultation rates, and the proportion of visits attributable to malaria were all greater in the facilities visited in Dubréka, on average. Within Conakry, only the facilities in Kaloum reported figures (167 and 179 cases per 1,000 residents) rivaling those seen routinely in Dubréka (range 167–578 cases/1,000) (Appendix Table 6; Appendix Figure 7).

The facility-reported data from both Dubréka and Conakry were found to be largely accurate when compared with registry records. All clinical sites in the study produced both surveillance reporting forms and clinical registers for comparison for all months requested. Of the 14 sites visited, 12 had reported malaria incidence data to within 10% of the true values listed in the clinical registers (Appendix Table 6, Appendix Figure 8). Notable discrepancies between the reported and registered sources of data were identified at 2 clinical sites. At both locations, both the reported number of consults and the reported number of malaria cases were incorrectly elevated during the period examined.

Potential Larval Habitat Enumeration

Potential mosquito larval habitats were sampled in 5 designated areas across Conakry and 2 linear village transects in Dubréka. A total of 187 potential larval habitats were identified within Conakry, and 28 were identified in Dubréka (Appendix Table 7; Appendix Figure 4). The types and proportions of larvae found in Conakry and Dubréka did not statistically differ from one another. No *Anopheles* larvae were found in Conakry; 1 *Anopheles* sp. larval habitat was identified in Dubréka. The most frequently identified mosquito larvae in both Conakry and Dubréka were *Culex* sp. (Appendix Table 7).

Discussion

Heterogeneity of Transmission

Using the data we collected, we can calculate a crude approximation of the relative effectiveness of nets as a disease preventative in the locations studied. The ratio of nets needed to provide universal coverage to the number of locally acquired incident cases would be much higher in Conakry (11:1) as compared to Dubréka (1.2:1), where incidence is several-folds higher and risk of acquiring malaria is greater. Similarly, within Conakry itself, we noted considerable heterogeneity of the risk of locally acquired malaria. Using the PAR to adjust for the risk of malaria associated with travel outside the city, the incidence of locally acquired, clinically diagnosed malaria is 1.79–3.08 times higher in Kaloum than that in the other 4 communes of Conakry. This heterogeneity in risk of local malaria infection could have profound implications for the distribution of mosquito nets in the future. For instance, recalculating the nets required for universal coverage of the population of Kaloum and the number of estimated locally acquired malaria cases gives a ratio of 3.1:1. Thus, these data can be used to not only ascertain the need for net distribution in Conakry, but also to prioritize portions of the city over others in the event the campaign is unable to provide universal coverage. Of note, these calculations rely on a number of assumptions that our rapid study cannot ensure, thereby reducing their overall precision and accuracy.

Limitations

While our study quickly gathered actionable information crucial to the NMCP for use during its net distribution campaign, it does have important limitations. In order to achieve results in a timely fashion, some generalizability had to be sacrificed. The findings presented here summarize a brief snapshot in time, and as such, cannot be used to make broad generalizations of malaria transmission throughout the year. Although we were able to demonstrate the likely presence of ongoing autochthonous malaria transmission in Conakry, estimations of the relative contribution of local transmission to urban disease burden are subject to a seasonal variability that we are unable to document here.

In addition to the temporal restrictions on the generalizability of our findings, the study populations in both Conakry and Dubréka are not representative of the underlying populations overall because they were purposefully selected. Not only were those in the geographic areas of greatest risk consciously selected, but children <5 years were overrepresented in the household testing. Odds ratios and comparisons as calculated are thus not reflective of Conakry and Dubréka overall but are rather comparisons of the subpopulations of each in the areas with the greatest disease burden. We purposefully biased our sample to increase our ability to detect the presence of low levels of ongoing

local malaria transmission at the expense of some degree of quantitative accuracy of the underlying population as a whole. More robust quantification of the risk of contracting malaria in Conakry requires more extensive sampling over a longer period.

Despite these limitations, our approach allowed us to describe the heterogeneity in urban disease burden and to provide an estimation of the risk of local disease acquisition in a relatively short period and within a relatively modest budget of $15,000. Moreover, in contrast to earlier studies, our study was conducted in the context of increasingly available routine data. Routine data were used to select study sites and were found to be generally reliable over the course of the study, thus providing evidence that routine data can be used for further monitoring of malaria risk in Conakry. The results of our study, while not broadly generalizable, yielded practical, actionable information in a timely fashion to directly influence use of disease prevention resources.

Additional References

1. Institut National de la Statistique. La Région de Conakry en chiffres. 2018 [cited 2020 Dec 29]. https://www.stat-guinee.org/images/Documents/Publications/INS/annuelles/annuaire/La%20Guine%20en%20chiffre%202018.pdf

2. Institut National de la Statistique. Enquête demographique et de santé et à indicateurs multiples (EDS-MICS 2012). 2013 [cited 2021 Jan 8]. https://dhsprogram.com/methodology/survey/survey-display-391.cfm

3. Institut National de la Statistique. Enquête par grappes à indicateurs multiples (MICS, 2016), Rapport final. 2017 [cited 2020 Dec 29]. https://dhsprogram.com/methodology/survey/survey-display-466.cfm

4. President's Malaria Initiative. Guinea malaria operational plan FY 2019. 2018 [cited 2020 Dec 29]. https://www.pmi.gov/docs/default-source/default-document-library/malaria-operational-plans/fy19/fy-2019-guinea-abbreviated-malaria-operational-plan.pdf

5. World Health Organization. World malaria report 2018. Report no. CC BY-NC-SA 3.0 IGO. 2018 [cited 2020 Dec 29]. https://www.who.int/malaria/publications/world-malaria-report-2018/en

6. Robert V, Macintyre K, Keating J, Trape J-F, Duchemin J-B, Warren M, et al. Malaria transmission in urban sub-Saharan Africa. Am J Trop Med Hyg. 2003;68:169–76. PubMed

7. Kouassi BL, de Souza DK, Goepogui A, Balde SM, Diakité L, Sagno A, et al. Low prevalence of *Plasmodium* and absence of malaria transmission in Conakry, Guinea: prospects for elimination. Malar J. 2016;15:175. PubMed

8. Labbo R, Fandeur T, Jeanne I, Czeher C, Williams E, Arzika I, et al. Ecology of urban malaria vectors in Niamey, Republic of Niger. Malar J. 2016;15:314. PubMed

9. Lekweiry KM, Ould Ahmedou Salem MS, Cotteaux-Lautard C, Jarjaval F, Marin-Jauffre A, Bogreau H, et al. Circumsporozoite protein rates, blood-feeding pattern and frequency of knockdown resistance mutations in *Anopheles* spp. in two ecological zones of Mauritania. Parasit Vectors. 2016;9:268. PubMed

10. Machault V, Gadiaga L, Vignolles C, Jarjaval F, Bouzid S, Sokhna C, et al. Highly focused anopheline breeding sites and malaria transmission in Dakar. Malar J. 2009;8:138. PubMed

11. Mourou JR, Coffinet T, Jarjaval F, Cotteaux C, Pradines E, Godefroy L, et al. Malaria transmission in Libreville: results of a one-year survey. Malar J. 2012;11:40. PubMed

12. Mwakalinga VM, Sartorius BK, Mlacha YP, Msellemu DF, Limwagu AJ, Mageni ZD, et al. Spatially aggregated clusters and scattered smaller loci of elevated malaria vector density and human infection prevalence in urban Dar es Salaam, Tanzania. Malar J. 2016;15:135. PubMed

13. Mudhune SA, Okiro EA, Noor AM, Zurovac D, Juma E, Ochola SA, et al. The clinical burden of malaria in Nairobi: a historical review and contemporary audit. Malar J. 2011;10:138. PubMed

14. Ferrari G, Ntuku HM, Ross A, Schmidlin S, Kalemwa DM, Tshefu AK, et al. Identifying risk factors for *Plasmodium* infection and anaemia in Kinshasa, Democratic Republic of Congo. Malar J. 2016;15:362. PubMed

15. Ferrari G, Ntuku HM, Schmidlin S, Diboulo E, Tshefu AK, Lengeler C. A malaria risk map of Kinshasa, Democratic Republic of Congo. Malar J. 2016;15:27. PubMed

16. Kazembe LN, Mathanga DP. Estimating risk factors of urban malaria in Blantyre, Malawi: a spatial regression analysis. Asian Pac J Trop Biomed. 2016;6:376–81.

17. Ngom R, Siegmund A. Urban malaria in Africa: an environmental and socio-economic modelling approach for Yaoundé, Cameroon. Nat Hazards. 2010;55:599–619.

18. Padilla JC, Chaparro PE, Molina K, Arevalo-Herrera M, Herrera S. Is there malaria transmission in urban settings in Colombia? Malar J. 2015;14:453. PubMed

19. Parizo J, Sturrock HJW, Dhiman RC, Greenhouse B. Spatiotemporal analysis of malaria in urban Ahmedabad (Gujarat), India: identification of hot spots and risk factors for targeted intervention. Am J Trop Med Hyg. 2016;95:595–603. PubMed

20. Rabarijaona LP, Ariey F, Matra R, Cot S, Raharimalala AL, Ranaivo LH, et al. Low autochthonous urban malaria in Antananarivo (Madagascar). Malar J. 2006;5:27. PubMed

21. Wang SJ, Lengeler C, Smith TA, Vounatsou P, Cissé G, Diallo DA, et al. Rapid urban malaria appraisal (RUMA) in sub-Saharan Africa. Malar J. 2005;4:40. PubMed

22. Wang SJ, Lengeler C, Smith TA, Vounatsou P, Cissé G, Tanner M. Rapid Urban Malaria Appraisal (RUMA) III: epidemiology of urban malaria in the municipality of Yopougon (Abidjan). Malar J. 2006;5:29. PubMed

23. Wang SJ, Lengeler C, Smith TA, Vounatsou P, Diadie DA, Pritroipa X, et al. Rapid Urban Malaria Appraisal (RUMA) I: epidemiology of urban malaria in Ouagadougou. Malar J. 2005;4:43. PubMed

24. Wang S-J, Lengeler C, Mtasiwa D, Mshana T, Manane L, Maro G, et al. Rapid Urban Malaria Appraisal (RUMA) II: epidemiology of urban malaria in Dar es Salaam (Tanzania). Malar J. 2006;5:28. https://doi.org/10.1186/1475-2875-5-28

25. Wang S-J, Lengeler C, Smith TA, Vounatsou P, Akogbeto M, Tanner M. . Rapid Urban Malaria Appraisal (RUMA) IV: epidemiology of urban malaria in Cotonou (Benin). Malar J. 2006;5:45. https://doi.org/10.1186/1475-2875-5-45

26. Thwing JI, Mihigo J, Fernandes AP, Saute F, Ferreira C, Fortes F, et al. How much malaria occurs in urban Luanda, Angola? A health facility-based assessment. Am J Trop Med Hyg. 2009;80:487–91. PubMed

27. Macedo de Oliveira A, Mutemba R, Morgan J, Streat E, Roberts J, Menon M, et al. Prevalence of malaria among patients attending public health facilities in Maputo City, Mozambique. Am J Trop Med Hyg. 2011;85:1002–7. PubMed

28. World Health Organization. Expanded programme on immunization. training for mid-level managers, module 7: the EPI coverage survey. 2008 [cited 2020 Dec 29]. https://www.who.int/immunization/documents/MLM_module7.pdf

29. World Health Organization. Guidelines for the treatment of malaria—3rd edition. 2015. https://www.who.int/publications/i/item/9789241549127

30. Mwangungulu SP, Sumaye RD, Limwagu AJ, Siria DJ, Kaindoa EW, Okumu FO. Crowdsourcing vector surveillance: using community knowledge and experiences to predict densities and distribution of outdoor-biting mosquitoes in rural Tanzania. PLoS One. 2016;11:e0156388. https://journals.plos.org/plosone/article?id=10.1371/journal.pone.0156388

Appendix Table 1. Characteristics of households and household members visited during an entomological–epidemiologic survey of urban transmission in Conakry, Guinea, 2018*

	Conakry						
Characteristic	Kaloum	Dixinn	Matam	Matoto	Ratoma	Total	Dubréka
No. households	60	60	60	60	60	300	120
No. people	366	376	480	448	494	2164	919
Average occupancy	6.1	6.3	8.0	7.5	8.3	7.2	7.7
Average age, y	24.1	22.3	22.2	20.8	19.4	21.6	20.3
% <5 y of age	14.2	20.7	20.6	17.2	17.4	18.1	22.2
% Pregnant women	2.7	1.9	1.9	0.9	1.8	1.8	3.0
No. persons tested by RDT	205	217	229	219	232	1,102	451
No. sleeping spaces documented	173	169	248	206	220	1,016	486
No. persons/sleeping space	2.1	2.2	2.0	2.2	2.3	2.1	1.9

*Dubréka population served as rural control. RDT, rapid diagnostic testing.

Appendix Table 2. Characteristics of persons tested for *Plasmodium falciparum* malaria infection, Guinea, 2018*

	Conakry								
Characteristic, n/N (%)	Kaloum	Dixinn	Matam	Matoto	Ratoma	Total	p value†	Dubréka	p value‡
No. persons tested by RDT	205	217	229	219	232	1102		451	
Persons <5 y tested, %	22.0	30.6	36.2	31.1	28.4	29.9	0.031	35.0	0.047
Positivity by RDT, <5 y	5/45 (11.1)	2/67 (3.0)	2/83 (2.4)	1/68 (1.5)	4/66 (6.1)	14/329 (4.3)	0.13	60/158 (38.0)	<0.001
Positivity by RDT, ≥ 5 y	17/160 (10.6)	8/150 (5.3)	1/146 (0.7)	4/151 (2.6)	13/166 (7.8)	43/773 (5.6)	<0.001	82/293 (28.0)	<0.001
Positivity by RDT, pregnant women	3/9 (33.3)	0/6 (0.0)	0/9 (0.0)	0/4 (0.0)	3/8 (37.5)	6/36 (16.7)	0.079	2/24 (8.3)	0.46
No history of travel outside Conakry for ≥1 y	154/205 (75.1)	127/217 (58.5)	143/229 (62.4)	148/219 (67.6)	145/232 (62.5)	717/1,102 (65.1)	NA	NA	NA
RDT+	17/154 (11.0)	2/127 (1.6)	1/143 (0.7)	2/148 (1.4)	7/145 (4.8)	29/717 (4.0)	<0.001	NA	NA

*In a joint epidemiologic–entomologic investigation of urban malaria transmission in Guinea, participants were tested by rapid diagnostic test (RDT) during community screening. NA, not applicable.
†Comparison of results for communes within Conakry.
‡Comparison of results for Conakry and Dubréka

Appendix Table 3. Factors associated with testing positive by RDT during entomological-epidemiologic assessment of urban transmission in Conakry, 2018*

Factor	Coefficients	Odds ratio	95% CI	p value	RDT positivity within group, n/N (%)
Fixed effects					
	District				
	Matam	Reference			3/229 (1.3)
	Dixinn	3.329	(0.78,18.18)	0.12	10/217 (4.6)
	Matoto	2.217	(0.43,13.28)	0.34	5/219 (2.3)
	Ratoma	6.795	(1.76,36.15)	0.0099	17/232 (7.3)
	Kaloum	13.968	(3.63,78.83)	0.00052	22/205 (10.7)
	Age, y				
	0–4	Reference			14/329 (4.3)
	5–14	0.987	(0.41,2.35)	0.98	16/278 (5.8)
	15–29	0.859	(0.33,2.21)	0.75	21/254 (8.3)
	≥30	0.185	(0.05,0.57)	0.0054	6/241 (2.5)
	Last time outside Conakry				
	Never left Conakry	Reference			10/404 (2.5)
	Within last 4 wk	7.269	(2.48,23.17)	0.00041	14/145 (9.7)
	4 wk–6 mo	4.990	(1.53,16.19)	0.0068	9/110 (8.2)
	6 mo–1 y	2.232	(0.56,8.09)	0.23	5/128 (3.9)
	>1 y	2.809	(1.07,7.84)	0.039	19/313 (6.1)
	Other:pregnancy				
	Denied pregnancy	Reference			51/1,065 (4.8)
	Pregnant	3.852	(0.95,15.79)	0.056	6/36 (16.7)
	Other: used LLIN				
	Did not sleep under net last night	Reference			51/902 (5.7)
	Slept under net last night	0.561	(0.17,1.58)	0.30	6/200 (3.0)
Random effects	Intercept grouped by household†		(0.54,2.48)		

*LLIN, long-lasting insecticidal net; RDT, rapid diagnostic test.
†SD = 1.411.

Appendix Table 4. Malaria test results and self-disclosed travel history <4 wk before seeking medical attention for suspected malaria, Conakry, December 2018

Characteristic	Conakry						p value
	Kaloum	Dixinn	Matam	Matoto	Ratoma	Total	
No. patients tested	181*	222	729	1,107	2,440	4,678	
No. blood smears	0	0	12	1†	18	31†	
No. RDTs	181*	222	717	1,107	2,422	4,648	
Confirmed positive cases, n/N (%)	116/180 (64.4)	53/222 (23.9)	149/729 (20.4)	273/1,107 (24.7)	765/2,440 (31.4)	1,356/4,678 (29.0)	$<2.2 \times 10^{-16}$
Positive travel history in patients testing positive, n/N (%)	55/116 (47.4)	7/53 (13.2)	31/149 (20.8)	55/273 (20.1)	69/765 (9.0)	217/1,356 (16.0)	$<2.2 \times 10^{-16}$
Positive overall travel history, n/N (%)	66/180 (36.7)	8/222 (3.6)	50/729 (6.9)	138/1,107 (12.5)	114/2,440 (4.7)	376/4,678 (8.0)	$<2.2 \times 10^{-16}$
Positive RDT result in patients who traveled, n/N (%)	55/66 (83.3)	7/8 (87.5)	31/50 (62.0)	55/138 (39.9)	69/114 (60.5)	217/376 (57.7)	
Relative risk of traveling outside Conakry	1.56	4.07	3.57	1.77	2.02	2.18	
Population-attributable fraction of risk associated with recent travel, %	17.0	10.0	15.0	8.8	4.6	8.7	

*One record was excluded from travel analysis due to contradicting, duplicate entry indicated a simultaneous positive and negative travel history. RDT, rapid diagnostic test.
†One patient received both a blood smear for microscopy and RDT.

Appendix Table 5. Severe malaria criteria in addition to a laboratory confirmation, as defined by the World Health Organization, 2015*

Symptom	Description
Impaired consciousness	Glasgow score <11 in adults or a Blantyre coma score <3 in children
Prostration	Generalized weakness such that patient is unable to sit, stand, or walk without assistance
Multiple convulsions/seizures	>2 episodes within 24 h
Acidosis	A base deficit >8 mEq/L, or plasma bicarbonate level <15 mmol/L, or venous plasma lactate $\geq$5 mmol/L. Severe acidosis manifests clinically as respiratory distress
Hypoglycemia	Blood or plasma glucose <2.2 mmol/L (<40mg/dL)
Anemia	Hemoglobin concentration $\leq$5 g/dL or hematocrit $\leq$15% in children <12 years of age with *P. falciparum* parasite count >10 000/uL; hemoglobin concentration <7 g/dL or hematocrit <20% in adults WITH *P. falciparum* parasite count >10,000/uL
Renal Impairment	Plasma or serum creatinine concentration >265 µmol/L (>3 mg/dL) or blood urea concentration >20 mmol/L
Jaundice	Plasma or serum bilirubin concentration >50 µmol/L (>3 mg/dL) with *P. falciparum* parasite count >100,000/µL
Pulmonary edema	Radiographically confirmed OR O2 saturation <92% on room air with respiratory rate >30/min
Significant bleeding	Recurrent or prolonged bleeding from the nose, gums, or venipuncture sites; hematemesis or melena
Shock	Compensated shock is defined as capillary refill $\geq$3 seconds or temperature gradient on leg (mid to proximal limb), but no hypotension. Decompensated sock is defined as systolic blood pressure <70 mm Hg in children or <80 mm Hg in adults, with evidence of impaired perfusion (cool peripheries or prolonged capillary refill)
Hyperparasitemia	*P. falciparum* parasitemia >10%

*Definition taken from the 2015 World Health Organization Guidelines for the Treatment of Malaria, 3rd edition: a laboratory-confirmed case of malaria (i.e., confirmed by light microscopy visualization of parasites in patient blood smear or immunologic detection of parasite antigen with RDT) with any one of the symptoms listed.

Appendix Table 6. Healthcare facility malaria indicators as reported by routine surveillance (left) compared with figures verified in clinical registers during rapid epidemiologic–entomologic investigation (right), July–September 2018

	Reported by routine surveillance			Reported cases as % of confirmed cases in registers*		
Location	All-cause incidence	Malaria incidence	% Malaria	All-cause	Confirmed malaria	% Malaria
Conakry						
Kaloum A	202	167	71	100	99	100
Kaloum B	358	179	50	101	101	100
Dixinn A	445	79	18	101	101	100
Dixinn B	1	0.2	24	133	176	130
Matam A	315	50	16	103	101	98
Matam B	338	51	15	98	102	104
Matoto A	19	5	25	102	90	90
Matoto B	82	19	23	100	99	99
Ratoma A	13	4	30	115	176	150
Ratoma B	163	51	31	100	100	99
Dubréka						
Dubréka A	752	578	77	99	100	99
Dubréka B	601	448	75	100	100	99
Dubréka C	369	212	57	100	100	100
Dubréka D	304	167	55	99	101	102

*Shading indicates a range of underreporting (deep turquoise) to overreporting (deep red) of data.

0–24%	25–49%	50–74%	75–99%	101–125%	126–150%	151–175%	>175%

Underreporting

Overreporting

Appendix Table 7. Key results of mosquito larval habitat searches conducted during rapid epidemiologic–entomologic investigation of urban malaria transmission, Guinea, 2018*

	Conakry								
Result	Kaloum	Dixinn	Matam	Matoto	Ratoma	Total	p-value†	Dubréka	p-value‡
Total potential larval habitats sampled	35	34	28	40	50	187		28	
Area sampled, m^2§	89,983	49,287	83,343	72,912	94,737	390,262		NA	
1/Sampling density, m^{-2}	2,571	1,450	2,977	1,823	1,895	2,087		NA	
No. without mosquito larvae, n/N (%)	28/35 (80.0)	19/34 (55.9)	15/28 (53.6)	33/40 (82.5)	35/50 (70.0)	130/187 (69.5)	0.023	21/28¶ (75.0)	0.71
No. with *Anopheles* larvae n/N (%)	0/35 (0.0)	0/34 (0.0)	0/28 (0.0)	0/40 (0.0)	0/50 (0.0)	0/187 (0.0)		1/28¶ (3.6)	0.27
No. with *Culex* larvae n/N (%)	7/35 (20.0)	13/34 (38.2)	9/28 (32.1)	7/40 (17.5)	12/50 (24.0)	48/187 (25.7)	0.24	5/28¶ (17.9)	0.51
1/*Culex* larval site density (m^{-2})	12,855	3,791	9,260	10,416	7,895	8,130		NA	
No. with *Aedes* larvae n/N (%)	0/35 (0.0)	2/34 (5.9)	4/28 (14.3)	0/40 (0.0)	3/50 (6.0)	9/187 (4.8)	0.049	3/28¶ (10.7)	0.41
1/*Aedes* larval site density (m^{-2})	0	24,644	20,834	0	31,579	43,362		NA	

*NA, not applicable.
†Comparison of results for communes within Conakry.
‡Comparison of results for Conakry and Dubréka.
§Area sampled estimated using Google Maps (see Appendix figure 1). Larval habitats in Dubréka sampled via transect, precluding calculation of density.
¶Numerators do not add to denominator due to larval habitats containing multiple genera.

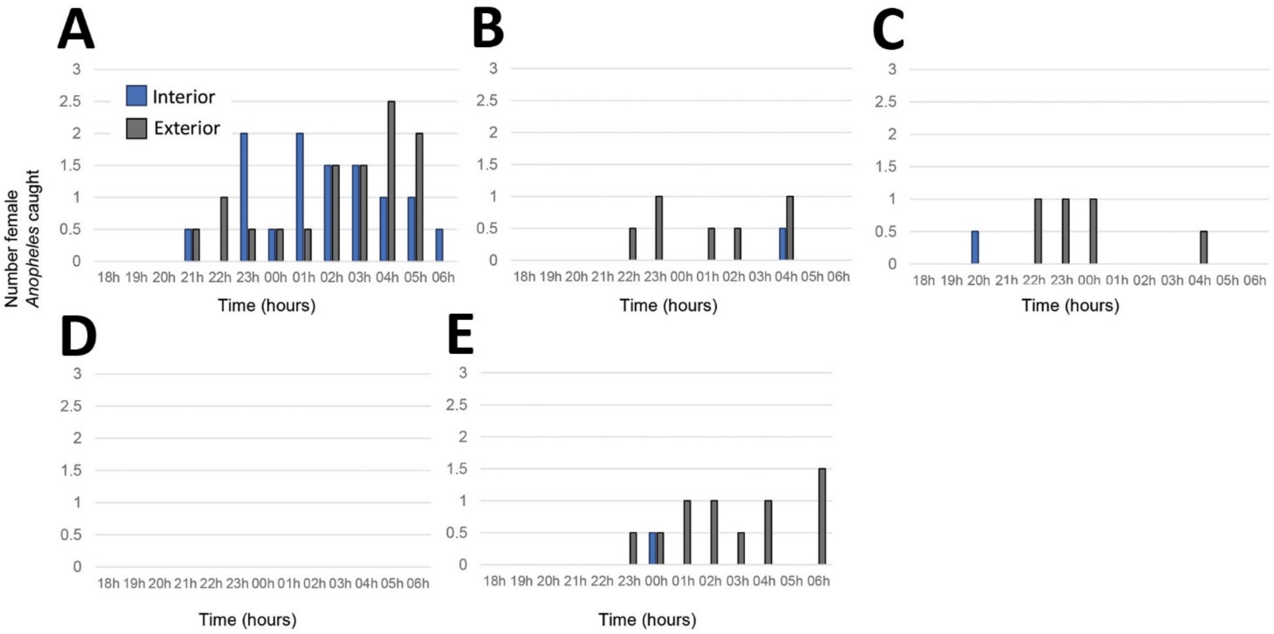

Appendix Figure 1. Average hourly adult female *Anopheles* captured by human landing catches, Conakry, Guinea, 2018. Adult female *Anopheles* mosquitoes were captured for 2 consecutive nights in each commune, with each night simultaneously consisting of indoor (blue bars) and outdoor (dark gray bars) captures. Data are shown for 5 communes of Conakry: Kaloum (A); Dixinn (B); Matam (C); Matoto (D); Ratoma (E).

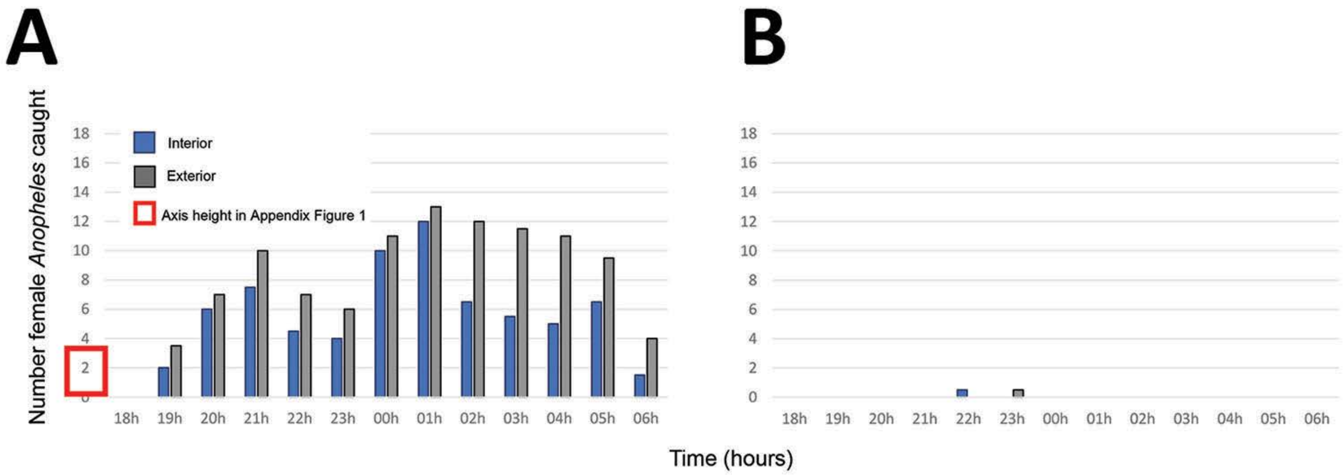

Appendix Figure 2. Average hourly adult female *Anopheles* captured by human landing catches, Dubréka, 2018. Adult female *Anopheles* mosquitoes were collected for 2 consecutive nights in each selected site, with each night simultaneously consisting of indoor (blue bars) and outdoor (dark gray bars) captures. Data are shown for 2 villages in Dubréka: Gbantama (A) and Falessade (B).

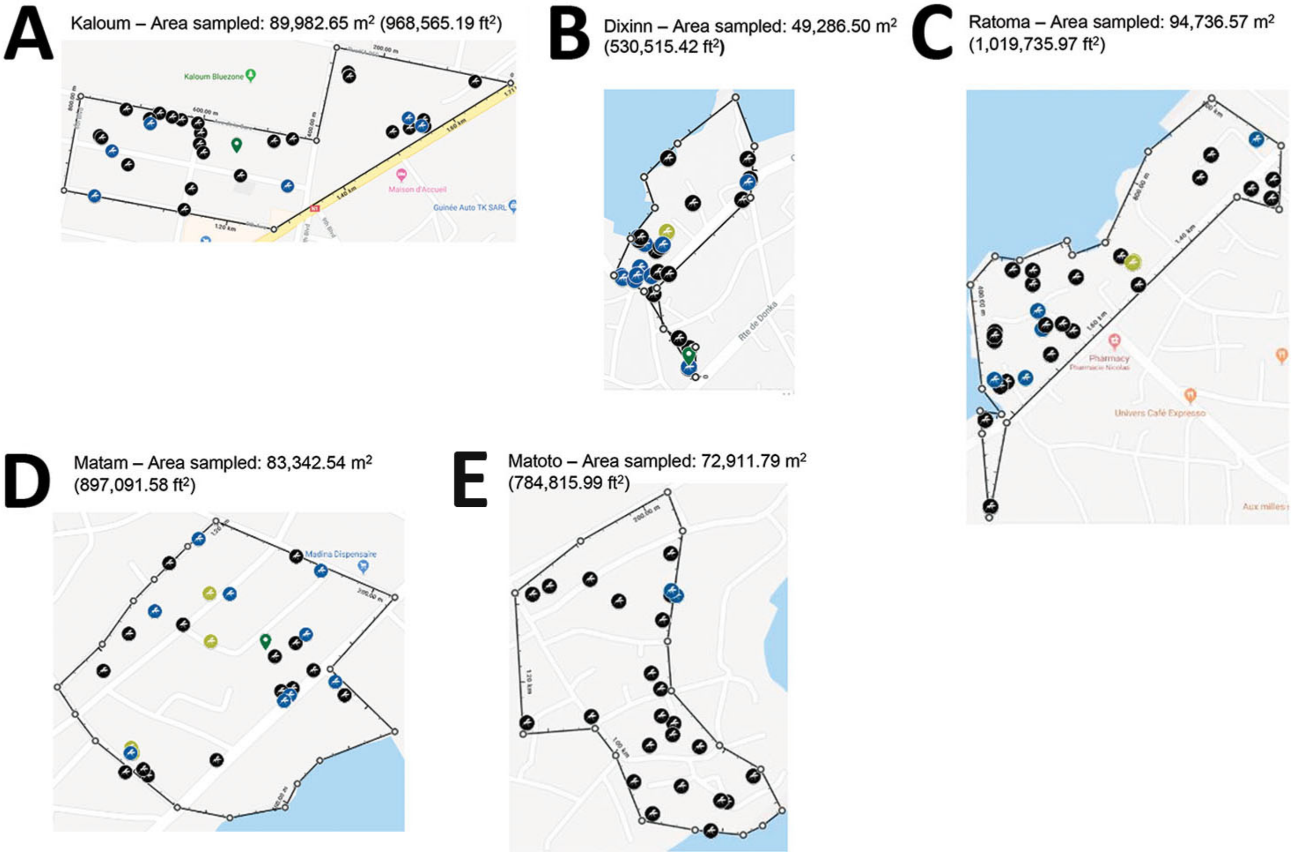

Appendix Figure 3. Urban search areas used for mosquito larval habitat sampling during combined epidemiologic–entomologic investigation, Conakry, Guinea, 2018. Polygons demarcate borders of the zones defined for the exhaustive search for potential larval habits. Mosquito icons denote the presence of collections of standing water, with the color representing mosquito larvae present: black, none; blue, *Culex*; yellow, *Aedes;* red, *Anopheles.* Data are shown for 5 communes of Conakry: Kaloum (A); Dixinn (B); Matam (C); Matoto (D); Ratoma (E).

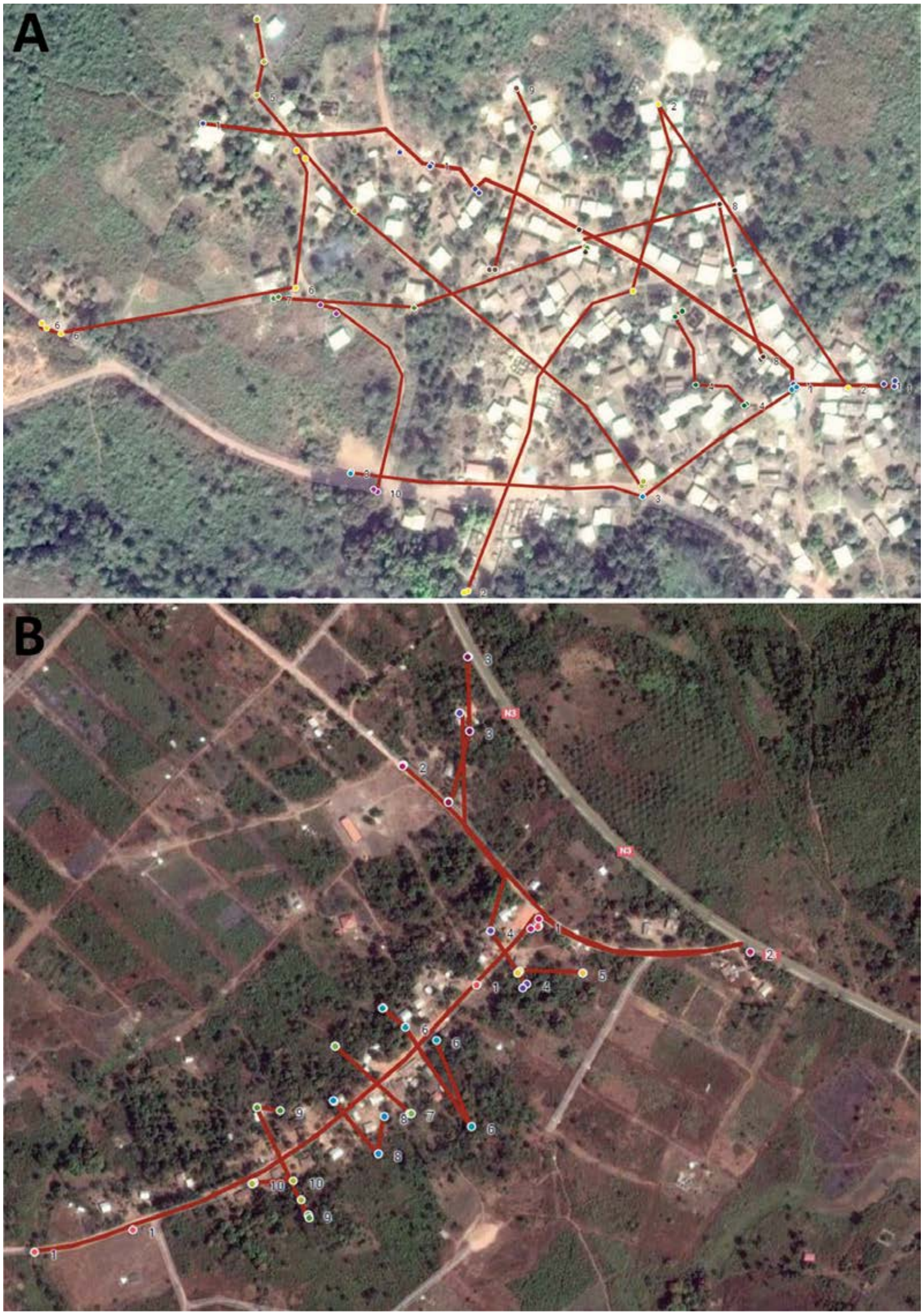

Appendix Figure 4. Village transects used for mosquito larval habitat sampling during combined epidemiologic/entomological investigation, Dubréka, Guinea, 2018. A) Transects used on December 3, 2018, Falessade; 3.230 km total. B) Transects used on December 4, 2018, Gbantama, 2.815 km total. Red lines represent transects walked during the search for potential larval habits. Locations of potential larval habits are not pictured.

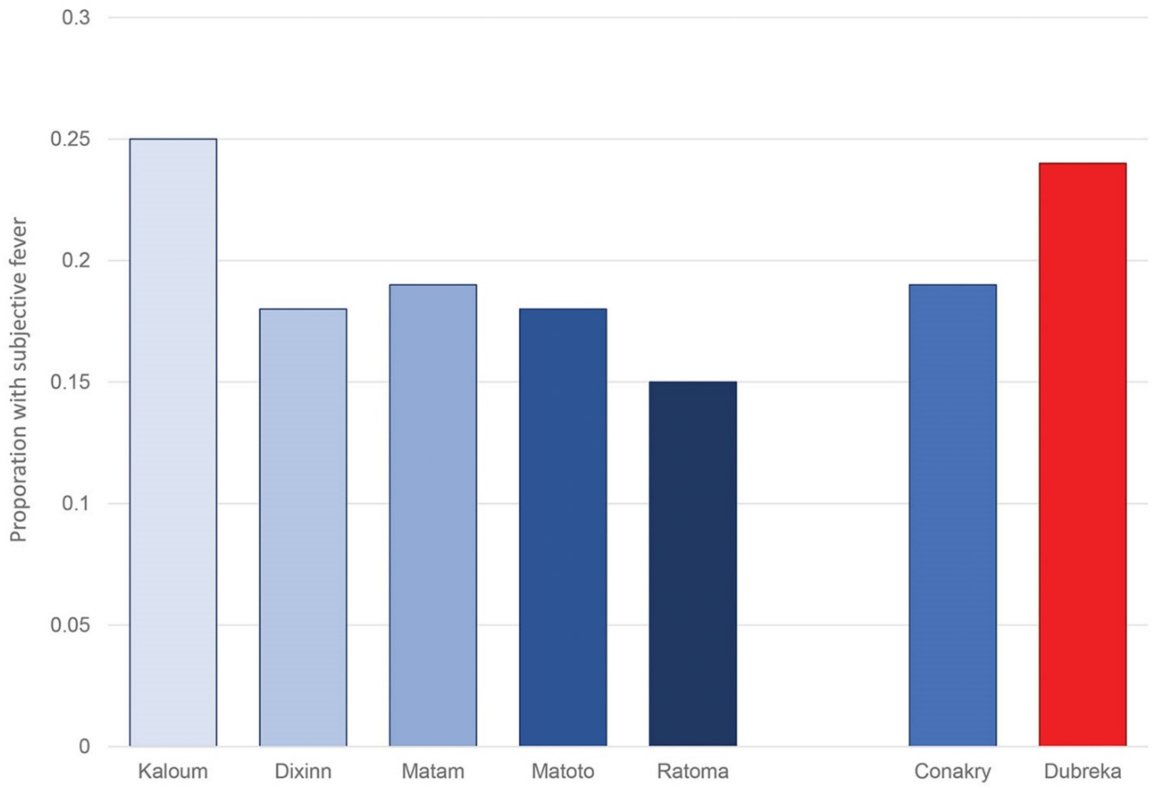

Appendix Figure 5. Proportion of respondents reporting fever within last 2 weeks as assessed by household questionnaire during combined epidemiologic–entomologic investigation of urban malaria transmission, Guinea, 2018**.** Results by commune of Conakry are depicted on the left, and overall estimates for Conakry and Dubréka on the right.

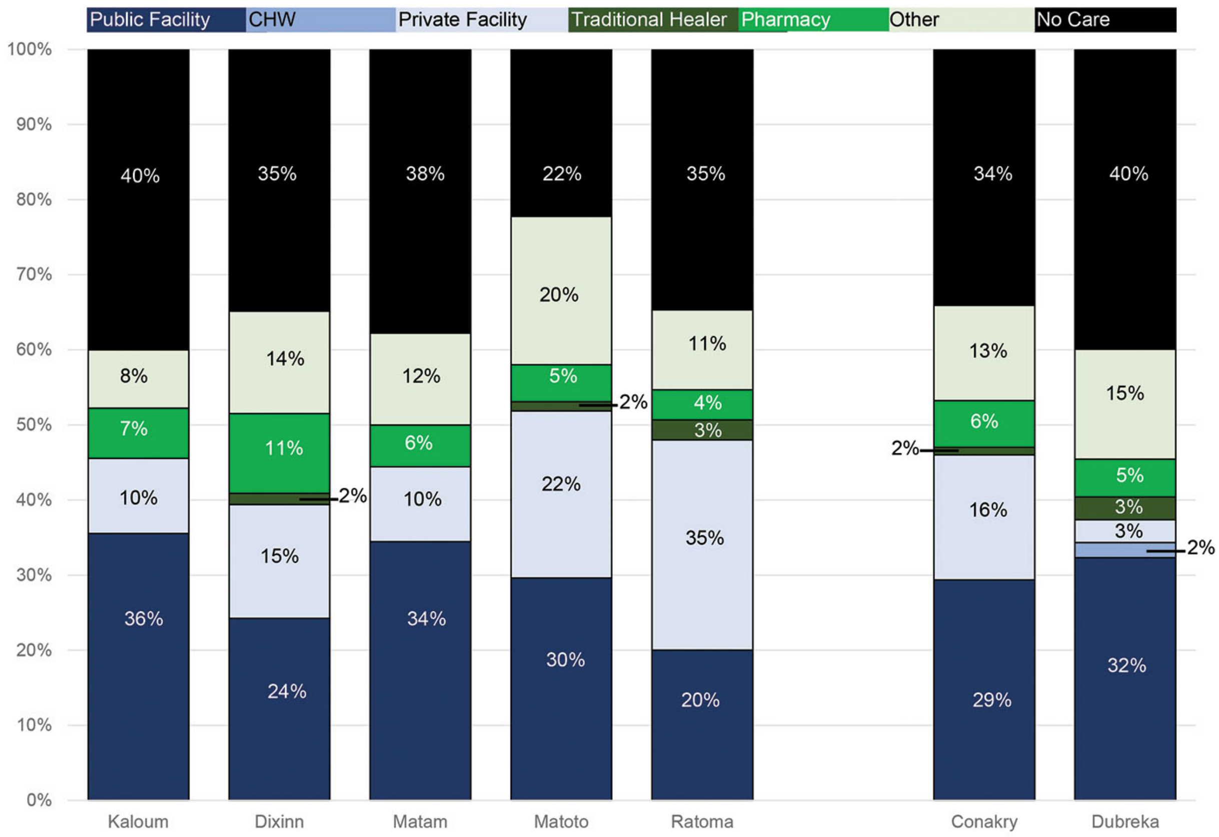

Appendix Figure 6. Distribution of persons seeking healthcare for fever in last 2 weeks as determined by self-report during rapid epidemiologic–entomologic investigation in Conakry and Dubréka, Guinea, 2018. Distributions of individual urban communes of Conakry are presented on the left; distributions of Conakry as a whole and Dubréka are on the right.

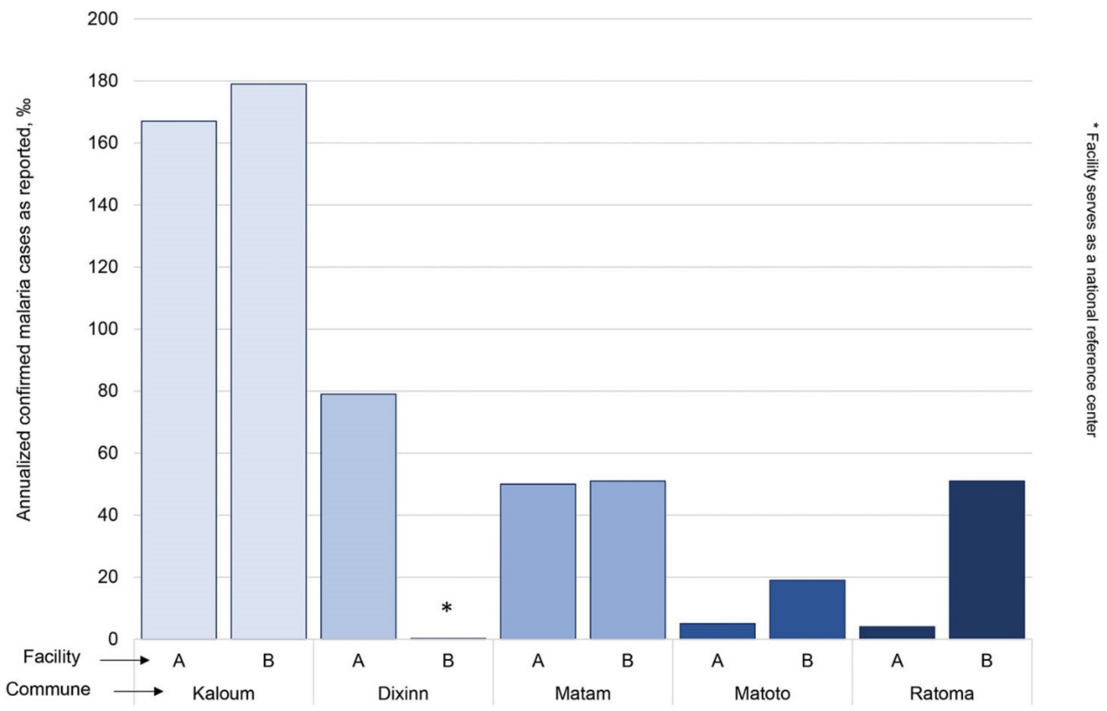

Appendix Figure 7. Malaria incidence in selected healthcare facilities in Conakry, Guinea, July–September 2018. Data were reported by routine surveillance and annualized per 1,000 population.

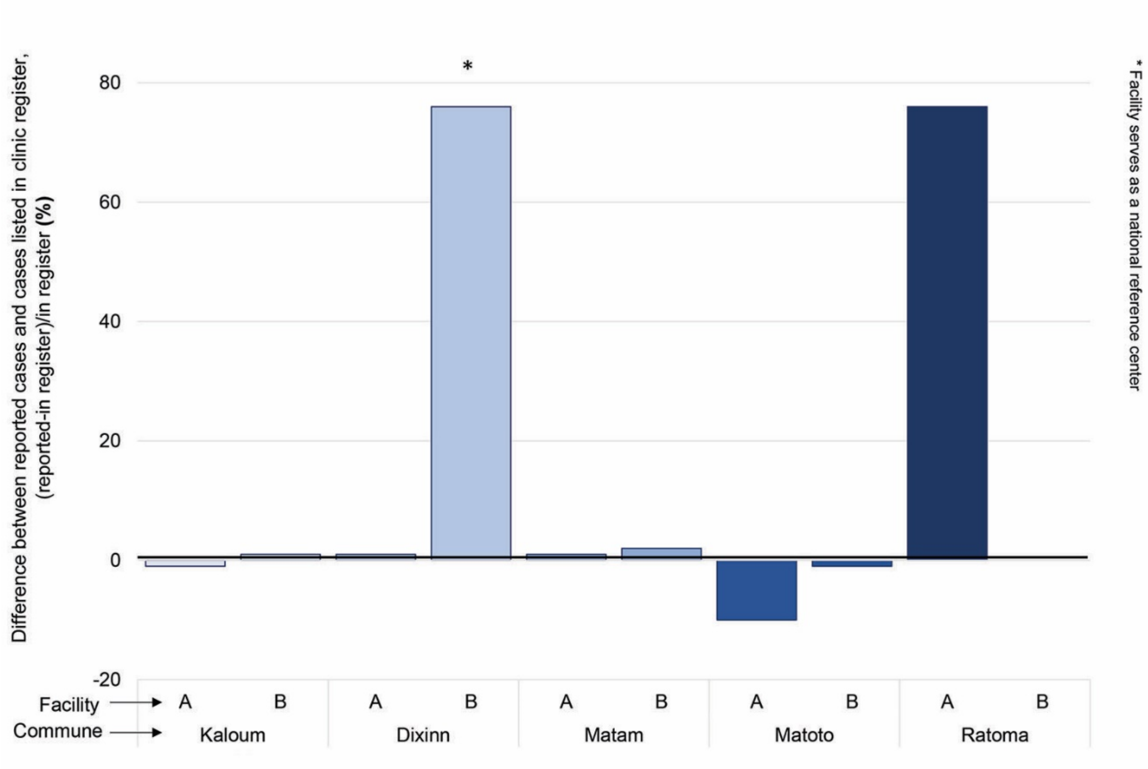

Appendix Figure 8. Comparison of health facility reported cases of malaria by routine surveillance and those listed in clinical registers, Conakry, Guinea, July –September 2018.
